# Neighborhood Deprivation and DNA Methylation and Expression of Cancer Genes in Breast Tumors

**DOI:** 10.1001/jamanetworkopen.2023.41651

**Published:** 2023-11-06

**Authors:** Brittany D. Jenkins, Emily Rossi, Catherine Pichardo, William Wooten, Margaret Pichardo, Wei Tang, Tiffany H. Dorsey, Anuoluwapo Ajao, Ruby Hutchison, Leah Moubadder, Lauren E. McCullough, Maeve Bailey-Whyte, Stefan Ambs

**Affiliations:** 1 Laboratory of Human Carcinogenesis, Center for Cancer Research, National Cancer Institute, Bethesda, Maryland; 2 Division of Cancer Control and Population Sciences, National Cancer Institute, Rockville, Maryland; 3 Department of Biostatistics, University of Maryland School of Medicine, Baltimore, Maryland; 4 Department of Surgery, Hospital of the University of Pennsylvania, Philadelphia, Pennsylvania; 5 Data Science & Artificial Intelligence, R&D, AstraZeneca, Gaithersburg, Maryland; 6 School of Medicine, Case Western Reserve University, Cleveland, Ohio; 7 Department of Epidemiology, Rollins School of Public Health, Emory University, Atlanta, Georgia; 8 School of Medicine, University of Limerick, Limerick, Ireland

## Abstract

**Importance:**

The biological processes that underlie the association of neighborhood environment with chronic diseases, such as cancer, remain poorly understood.

**Objective:**

To determine whether differences in breast tissue DNA methylation are associated with neighborhood deprivation among Black and White women with breast cancer.

**Design, Setting, and Participants:**

This cross-sectional study collected breast tissue from women undergoing surgery for breast cancer between January 1, 1993, and December 31, 2003. Participants were recruited through the University of Maryland Medical Center, with additional collection sites at Baltimore-area hospitals. Data analysis was performed from March 1 through December 1, 2022.

**Exposure:**

Year 2000 census tract–level socioeconomic deprivation measured via neighborhood deprivation index (NDI) as a standardized score, with Black and White race being ascertained through self-report.

**Main Outcome and Measures:**

The primary outcome was tissue DNA methylation using genome-wide measurements. The secondary outcome was tissue gene expression.

**Results:**

Participants included 185 women with breast cancer (110 Black [59.5%], 75 White [40.5%]). Mean (SD) age at surgery was 56.0 (14.1) years. Neighborhood deprivation was higher for Black women than for White women (Mean [SD] NDI, 2.96 [3.03] for Black women and −0.54 [1.91] for White women; difference, −3.50; 95% CI, −4.22 to −2.79; *P* < .001). In unstratified analysis, 8 hypomethylated CpG sites were identified as associated with the NDI, including sites in 2 tumor suppressor genes, *LRIG1* and *WWOX*. Moreover, expression of the 2 genes inversely correlated with neighborhood deprivation. In the race-stratified analysis, the negative correlation between the *LRIG1* gene body CpG site cg26131019 and the NDI remained significant in Black women. A neighborhood deprivation–associated decrease in gene expression was also observed for *LRIG1* and *WWOX* in tumors from Black women.

**Conclusions and Relevance:**

In this study, high neighborhood deprivation was associated with differences in tissue DNA methylation and gene expression among Black women. These findings suggest that continued investment in public health interventions and policy changes at the neighborhood level may help to remedy biological alterations that could make minoritized populations more susceptible to chronic diseases.

## Key Points

**Question:**

What is the association between neighborhood deprivation, DNA methylation, and gene expression in breast tissue for Black and White women with breast cancer?

**Findings:**

In a cross-sectional study of 185 women with breast cancer, higher neighborhood deprivation was associated with decreased methylation and gene expression of 2 tumor suppressor genes, *LRIG1* and *WWOX*, for Black patients with breast cancer.

**Meaning:**

These findings suggest that, for Black women, high neighborhood deprivation is associated with epigenetic differences in breast tumors that may lead to more aggressive disease, signaling the need for continued investment in public health interventions and policy changes at the neighborhood level.

## Teaser

This cross-sectional study examines differences in breast tumor DNA methylation among Black and White women with breast cancer living in areas of low and high neighborhood deprivation.

## Introduction

Recent studies have shown a decline in breast cancer mortality for every major racial and ethnic group.^1^ Still, a mortality gap exists between Black and White women, with Black women having a 40% higher mortality rate despite having lower overall breast cancer incidence rates.^1,2^ In investigating the social, environmental, and economic contributors to adverse health outcomes for minoritized communities across the US, several studies have assessed the association between neighborhood socioeconomic deprivation and health. Living in a deprived neighborhood can lead to adverse health outcomes, including higher rates of cancer^3,4,5^ and overall mortality.^6,7^ Given that our environments have such a strong influence on health,^8,9,10^ it is important that we understand the role of neighborhood-level exposures in the origin and outcome of cancer.

Few studies have sought to uncover the biological mechanisms that mediate the association between neighborhood environment and disease.^11,12,13,14^ One study investigated the association of contemporary redlining with DNA methylation in breast tumors and found redlining-associated methylation of CpG sites in genes previously linked to breast carcinogenesis.^13^ Functionally, these genes were related to inflammation, immune function, and stress response, which provides clues about how living in economically disadvantaged neighborhoods may influence health and cancer risk.^15,16^ Other studies that investigated differential DNA methylation in the context of neighborhood deprivation reported decreased global methylation associated with high deprivation^17^ in addition to differential expression of stress- and immune-related genes^18,19^ and changes in markers of epigenetic aging toward accelerated aging.^20,21^ These findings indicate that aberrant loss of methylation may be a candidate mediator of the association between neighborhood environment and health outcomes, especially for individuals living in deprived areas.

Other investigations have shown that epigenetic alterations associated with neighborhood deprivation may impact the expression of breast cancer–related genes.^22^ These findings are plausible, as a quantitative trait loci analysis investigating the link between DNA methylation at enhancers and gene expression across several breast cancer cohorts revealed that at least 2 gene regulatory networks were likely impacted by aberrant DNA methylation, one in estrogen receptor signaling and the other in immune cell infiltration.^23^ Additionally, the expression of the tumor suppressor gene *p53* is reduced in response to stress signals, promoting breast tumorigenesis.^24^ These studies and others allude to an epigenetics-driven link among neighborhood deprivation, the expression of cancer-related genes, and breast cancer development, yet additional evidence is needed in support of this pathway. Thus, we pursued the hypothesis that alterations in tumor DNA methylation may be associated with neighborhood deprivation among patients with breast cancer by mechanisms affecting cancer-related genes and the immune environment, thereby promoting breast cancer progression.

## Methods

### Study Population and Tissue Collection

This cross-sectional study included a convenience sample of 185 women with breast cancer (110 Black and 75 White) from a larger patient population of 456 women who donated tissue from breast surgery performed at the University of Maryland Medical Center, with additional collection sites at Baltimore-area hospitals. A description of the National Cancer Institute (NCI)-Maryland Breast Cancer Study, which follows the Strengthening the Reporting of Observational Studies in Epidemiology (STROBE) reporting guideline for observational studies, is provided in the eMethods in Supplement 1. This study was approved by the University of Maryland institutional review board and National Institutes of Health Office of Human Subjects Research Protections. All participants signed written informed consent and completed an interviewer-administered questionnaire.

The applied criteria to arrive at the final analytic population are described in the eMethods in Supplement 1. Tissue samples were collected mainly between January 1, 1993, and December 31, 2003 (132 participants [71%]). A small subset of additional participants was recruited after 2003 up until January 11, 2019 (53 participants [29%]). The study included US adults (aged 30-93 years) who self-reported as non-Hispanic Black (hereafter, Black) or non-Hispanic White (hereafter, White), which were the 2 major patient groups at the recruitment sites. Due to the social and economic implications of this work, terms describing race as a social construct (eg, Black or White) were used throughout.

### Neighborhood Deprivation Index

Neighborhood deprivation has historically been measured via a neighborhood deprivation index (NDI) to empirically summarize multiple census tract–level variables into a single standardized index for statistical analyses.^25^ We used an NDI adapted from Messer et al^25^ that has been described previously.^5^ Briefly, participant addresses were geocoded and linked to census tracts from the 2000 census using normalized data from the National Neighborhood Change Database produced by GeoLytics.^26^ A principal components analysis was used for data reduction based on a study that validated the index in Maryland^27^ (eMethods in Supplement 1). The following 6 variables were included in our index: percentage of households living in poverty, percentage of households receiving public assistance, percentage of female-headed households with dependent children, percentage of households earning less than $30 000 per year, percentage of males and females unemployed, and percentage of households with no car. Lower values indicate lower deprivation, while higher values indicate higher deprivation. For this analysis, the NDI was operationalized as either a continuous score, dichotomized at the median (less than or equal to the median vs greater than the median) or as quartiles with cutoffs based on distribution among women without breast cancer in our cohort (n = 104) (eFigure 1 in Supplement 1). We followed previous studies using different categorizations to assess neighborhood deprivation.^5,28^

### DNA Methylation

#### Preprocessing

To investigate genome-wide differences in DNA methylation in association with the NDI, we performed DNA extractions of breast tumor tissue using the DNeasy Blood and Tissue Kit (QIAGEN). DNA was sent to the NCI Genomics Technology Laboratory to be analyzed for DNA methylation at CpG sites using the Infinium MethylationEPIC 850K BeadChip (Illumina, Inc) according to the manufacturer’s protocol. DNA methylation in this analysis refers to β-values, which are continuous variables between 0 and 1, where 1 represents 100% methylation at the CpG site. β-Values represent the ratio of the intensity of the methylated (*M*) bead types to the total intensity of both methylated and unmethylated (*U*) bead types at each CpG site.^29^ They can be calculated using the equation β = [*M* / (*M* + *U*)].

#### Quality Control and Normalization

Normalization and quality control of the methylation data were completed using the DNAmArray package in R, version 4.2.2 (R Foundation for Statistical Computing). Quality control showed 6401 probes (0.7%) with a success rate of less than 0.95, which were therefore removed. On the basis of suggestions published by McCartney et al,^30^ we also filtered out 62 466 probes (7.2%) with a single-nucleotide variant (formerly single-nucleotide polymorphism) minor allele frequency of less than or equal to 0.05 in 1 of the studied populations. Additionally, 46 566 probes (5.3%) were removed that were previously identified as nonspecific. Our not applicable rate, which indicates the percentage of values below the limit of detection, was 0.0047%. We imputed these not applicable rates using the missMDA package in R to allow for functional normalization, which removes unwanted technical variation using control probes.^31^ Our approach yielded a total of 750 426 probes for the final analysis. Further details on methylation quality control are provided in the eMethods in Supplement 1.

### MethylCIBERSORT

Normalized methylation β-values were used for methylCIBERSORT deconvolution analysis to estimate immune cell subpopulation differences. Using the methylCIBERSORT R package^29,32^ and StromalMatrix_V2 as the methylation signature matrix, we determined the relative scores of 10 immune cell subpopulations. Analysis parameters included batch correction (B-mode), disabling quantile normalization, and 500 permutations per run.

### Statistical Analysis

Data analysis was performed from March 1 through December 1, 2022. All statistical testing was 2-sided. Associations were considered statistically significant with either an unadjusted *P* < .05 or an adjusted *P* value using the Holm method^33^ (threshold, 7.0 × 10^−8^) to account for multiple testing where appropriate. Student *t* and χ^2^ tests were used to assess associations of continuous and categorical variables, respectively, with the NDI. We used the CpGAssoc R package to compute differences in β-coefficients between continuous NDI measurements and individual CpG sites. Linear regression models were adjusted for potential confounding factors suggested in the literature, including age at surgery and self-reported race and tumor purity, where appropriate. *P* values and 95% CIs are reported. Analysis of variance tests were used to compare more than 2 categorical variables against a continuous variable. All tests used continuous methylation data unless otherwise noted. Data analyses were performed using Stata/SE, version 17.0 (StataCorp LLC); JMP, version 14.0 (SAS Institute, Inc); and R statistical software. For survival analysis, we assessed recurrence-free survival (RFS) and overall survival (OS) in reference to gene expression using the settings provided with the Kaplan-Meier Plotter (KMPlotter) webtool^34^ for breast cancer.^35^ Additional information on the study cohort, detailing exclusion criteria, race categorization, NDI analyses, quality control of methylation data, tumor purity variable, RNA sequencing preprocessing and analysis, and survival analysis are available in the eMethods in Supplement 1.

## Results

### Baseline Participant Characteristics

Our study consisted of 185 women with breast cancer from the Baltimore, Maryland, area (90 from low-deprivation neighborhoods and 95 from high-deprivation neighborhoods) who donated tumor tissue from breast surgery. The majority of women were Black (110 [59.5%] vs 75 White [40.5%]), had a high school education as their final academic degree (90 [48.6%]), and had an annual household income of $60 000 or less at the time of recruitment (121 [79.5%]). Mean (SD) age at surgery was 56.0 (14.1) years. Participant characteristics, including missing data, by dichotomized neighborhood status are shown in eTable 1 in Supplement 1. Neighborhood deprivation was higher among Black participants, those with an income of $60 000 or less, and those who were never married or were divorced, widowed, or separated (eTable 1 in Supplement 1).

### Association of Neighborhood Deprivation With Patient Group and Breast Cancer Molecular Subtypes

Neighborhood deprivation was different by self-reported race (Mean [SD] NDI, 2.96 [3.03] for Black women and −0.54 [1.91] for White women; difference, −3.50; 95% CI, −4.22 to −2.79; *P* < .001) (eFigure 2 in Supplement 1). On examination of dichotomized neighborhood deprivation by molecular subtypes, we observed that patients with triple-negative breast cancer (TNBC) comprised the highest proportion of individuals living in areas with high neighborhood deprivation, although this outcome was not statistically significant (eFigure 2 in Supplement 1).

### DNA Methylation and Gene Expression Differences in Cancer-Related Genes by Neighborhood Deprivation Status

**Figure 1.**
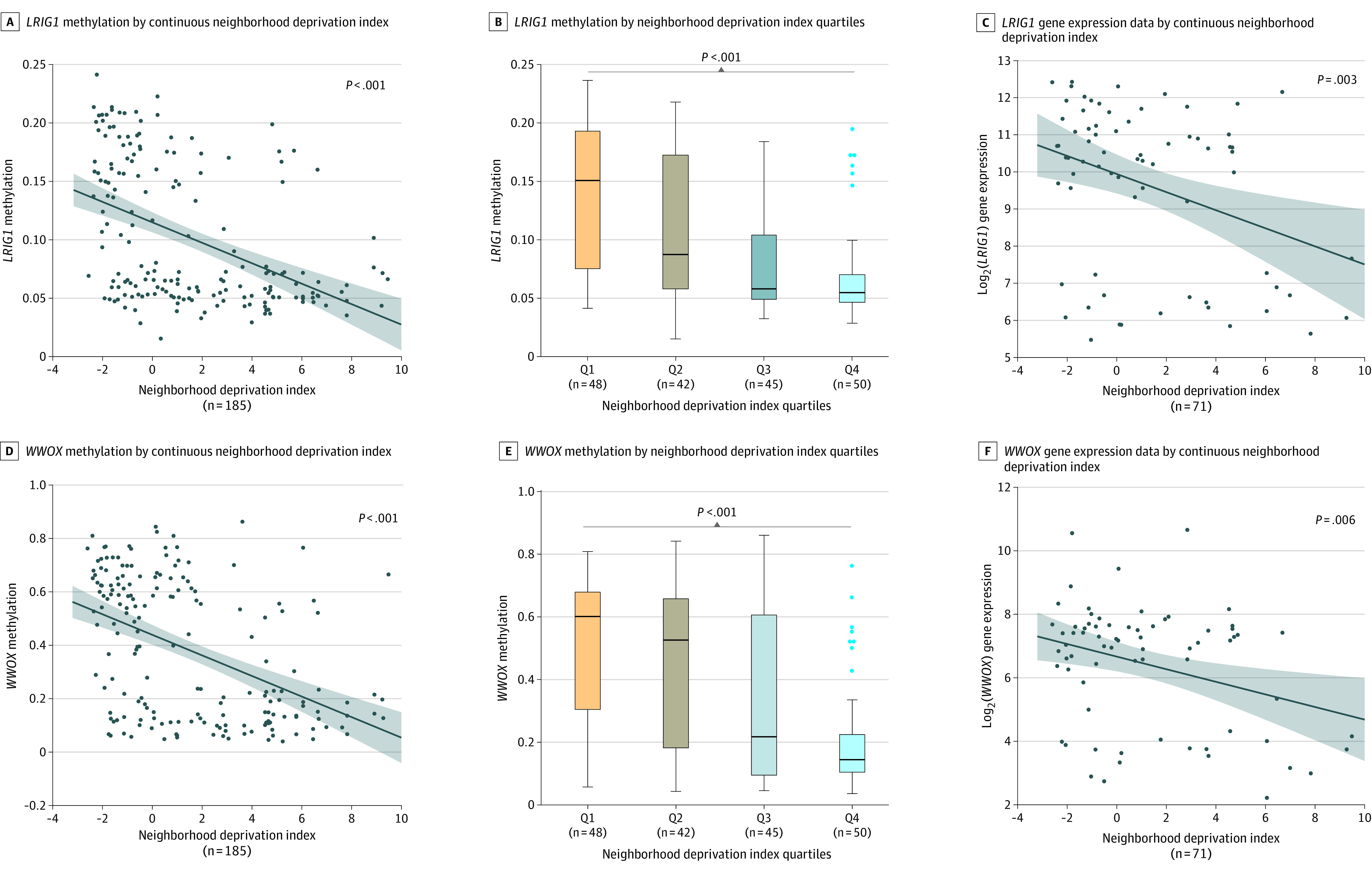
DNA Methylation and Gene Expression for the 2 Tumor Suppressor Genes *LRIG1* (Locus cg26131019) and *WWOX* (Locus cg02161209) by Neighborhood Deprivation In panels A, C, D, and F, the graphs depict a line of fit showing a linear regression with 95% CIs (shaded areas) for 2 continuous variables (x- and y-axes). In panels B and E, the horizonal bar inside the boxes indicates the median, and the lower and upper ends of the boxes are the 25th and 75th percentiles, respectively. The whiskers indicate the minimum and maximum values. *P* values were derived from the analysis of variance statistical test. Q indicates quartile.

We performed multiple linear regression models to assess the association between the NDI and methylation status at CpG sites with a genome-wide approach (n = 750 426), using the Holm method to account for multiple hypothesis testing. With the NDI coded as a continuous variable, we found 8 CpG sites to be associated with neighborhood deprivation after adjusting for age at surgery, tumor purity, and methylation batch (Table 1). Notably, 2 CpG sites located in the gene body region of tumor suppressor genes, namely *LRIG1* (leucine-rich repeats and immunoglobulin-like domains 1, cg26131019) and *WWOX* (WW domain–containing oxidoreductase, cg02171206), were among the associated loci in the unstratified analysis (Table 1; Figure 1). Because tumor RNA sequencing data were available for a subset of the patients (n = 71), we could include transcript levels for these 2 genes in our analyses. The expression of these 2 genes also showed an association with the NDI in which high neighborhood deprivation correlated with a decreased expression of these tumor suppressor genes (Figure 1C and F). Correlating gene promoter and body methylation with gene expression, the methylation status in both the promoter region (*r* = −0.24; 95% CI, −0.45 to 0.00; *P* = .046) and gene body region (*r* = −0.29; 95% CI, −0.49 to −0.06; *P* = .01) of *LRIG1* correlated with its transcript levels (eFigure 3 in Supplement 1).

**Table 1.** Associations Between Neighborhood Deprivation and CpG Site Methylation in Breast Tumors (n = 185) Using the CpGAssoc Package of R

CpG site	Reference gene^a^	Gene region of CpG site	Neighborhood deprivation^b^^,^^c^	
			*P* value	Adjusted *P* value^d^
cg13837834	*RNU6-1*	TSS1500	<.001	<.001
cg02171206	*WWOX*	Body	<.001	<.001
cg04118610	*LPHN3*	Body	<.001	<.001
cg26131019	*LRIG1*	First exon	<.001	<.001
cg15792220	*WWC3*	5′ UTR	<.001	<.001
cg16999677	*ZDHHC11*	TSS200	<.001	<.001
cg04399443	*ANKRD18CP*	3′ UTR	<.001	<.001
cg02380802	*RP11-44F14.2*	5′ UTR	<.001	<.001

^a^ Reference gene names and gene regions obtained from the Infinium MethylationEPIC 850K BeadChip (Illumina, Inc) annotation file and National Center for Biotechnology Information.

^b^ Neighborhood deprivation is on a continuous scale.

^c^ Linear regression model includes an adjustment for age at surgery, tumor purity, and methylation batch.

^d^ All data passed a significance threshold of 7.0 × 10^−8^ using the Holm method to account for multiple testing.

### Association of the *LRIG1* and *WWOX* Methylation and Expression Status With Neighborhood Deprivation Among Black Patients

**Figure 2.**
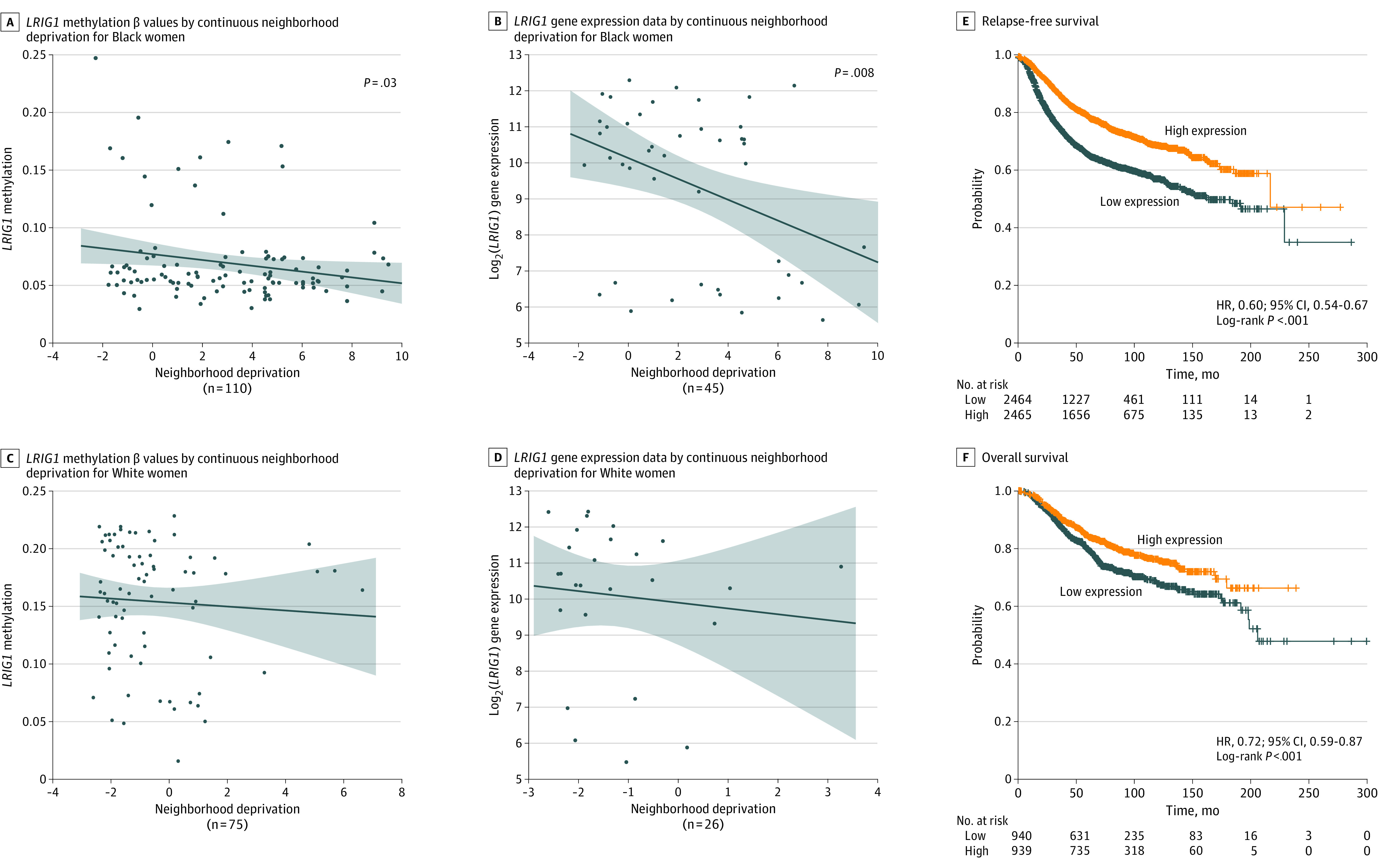
DNA Methylation and Gene Expression by RNA Sequencing of *LRIG1* (Locus cg26131019) by Continuous Neighborhood Deprivation Index, Stratified by Self-Reported Race In panels A-D, the graphs depict a line of fit showing a linear regression with 95% CIs (shaded areas) for 2 continuous variables (x- and y-axes). In panels E and F, the Kaplan-Meier plots are based on gene chip–derived expression data for *LRIG1* from breast tumors^35^ (Methods). HR indicates hazard ratio.

We continued our analysis with a focus on the CpGs located within the gene body of the 2 tumor suppressor genes, *LRIG1* and *WWOX.* While we did not adjust for race in our initial approach, since neighborhood deprivation and self-reported race were highly correlated in our cohort, we did stratify our more targeted analysis by patient group, investigating the association of *LRIG1* and *WWOX* methylation and gene expression with neighborhood deprivation. For *LRIG1*, we observed a significant negative correlation in Black patients (*r* = −0.20; 95% CI, −0.38 to −0.02; *P* = .03). *LRIG1* methylation decreased as neighborhood deprivation increased, a correlation we did not observe among White patients (Figure 2A and C; eTable 2 in Supplement 1). Similarly, *LRIG1* expression decreased when neighborhood deprivation increased, but only among the Black patients (*r* = −0.39; 95% CI, −0.61 to −0.11; *P* = .008) (Figure 2B and D; eTable 2 in Supplement 1). To explore whether *LRIG1* expression was associated with survival, we used the KMplotter webtool, with 4929 patients with breast cancer informative for RFS and 1879 patients informative for OS in the mRNA gene chip setting. *LRIG1* expression was inversely associated with both RFS and OS (Figure 2E and F). For RFS, significant associations were also found when the analysis was restricted to estrogen receptor–positive tumors (hazard ratio [HR], 0.57; 95% CI, 0.51-0.65) or to patients who either remained untreated following surgery (HR, 0.56; 95% CI, 0.45-0.69) or received adjuvant endocrine therapy (HR, 0.62; 95% CI, 0.47-0.81) (eFigure 4A-C in Supplement 1). Finally, the association with OS was replicated in the KMplotter and The Cancer Genome Atlas–based RNA sequencing data set, which does not provide RFS data (eFigure 4D in Supplement 1).

A similar analysis was performed for *WWOX* (eFigure 5A-D in Supplement 1). Here, the RNA sequencing analysis revealed an inverse association of *WWOX* gene expression with increasing neighborhood deprivation, which was significant among Black patients only (*r* = −0.43; 95% CI, −0.64 to −0.16; *P* = .003). No other associations were found.

### Differential Immune Profiles by Neighborhood Deprivation in Breast Tumors

We used methylCIBERSORT to estimate stromal and immune cell subpopulation differences in tumor tissues and stratified by dichotomized neighborhood deprivation. Using linear regression models, we found that for patients with breast cancer living in areas with a high NDI, the relative proportion of neutrophils was decreased compared with patients with breast cancer living in low NDI areas, after adjustment for age at surgery and self-reported race (β coefficient = −0.050; 95% CI, −0.082 to −0.018; *P* = .003) (Table 2; eFigure 6 in Supplement 1).

**Table 2.** Association of Neighborhood Deprivation With Abundance of Immune Cells in Breast Tumors After Applying MethylCIBERSORT and Linear Logistic Regression With Dichotomized Neighborhood Deprivation

Immune cell population	Neighborhood deprivation, adjusted model (185 tumors)^a^^,^^b^	
	β-Coefficient (95% CI)	*P* value
Regulatory T cells	−0.006 (−0.029 to 0.017)	.63
Neutrophils	−0.050 (−0.082 to −0.018)	.003
Fibroblasts	0.008 (−0.016 to 0.033)	.52
Eosinophils	0.003 (−0.012 to 0.019)	.70
Endothelial cells	0.007 (−0.003 to 0.017)	.18
CD8^+^ T cells	0.007 (−0.013 to 0.026)	.50
Natural killer cells (CD56)	0.005 (−0.001 to 0.011)	.08
Effector CD4^+^ T cells	0.002 (0.017 to 0.021)	.85
B cells (CD19)	0.003 (−0.010 to 0.017)	.63
Monocytes and macrophages (CD14)	0.020 (−0.002 to 0.042)	.07

^a^ Dichotomization of neighborhood deprivation was based on the median index values (less than or equal to the median vs greater than the median) in noncases (eMethods and eFigure 1 in Supplement 1).

^b^ Linear regression models adjusted for age at surgery and self-reported race.

## Discussion

In this cross-sectional study, we investigated whether neighborhood deprivation status is associated with methylation and gene expression differences in breast tumors of 185 women with breast cancer who self-reported as Black or White. To our knowledge, this study is the largest to examine differential methylation in breast tumors by neighborhood deprivation, with an overrepresentation of Black women relative to the general US population. Using a genome-wide discovery approach, our analysis revealed 8 CpG sites in which hypomethylation in tumors was positively correlated with increasing neighborhood deprivation, including CpG sites in 2 tumor suppressor genes, *LRIG1* and *WWOX*. In the race-stratified analysis, NDI was associated with both the methylation status and expression of *LRIG1* and *WWOX* among Black patients but not among White patients. We also found a lower proportion of neutrophils in tumors of patients residing in high neighborhood deprivation areas. Overall, however, our study revealed modest differences in DNA methylation relative to neighborhood deprivation and breast cancer disparities, providing needed insights into the extent of the association between deprivation and the more aggressive breast tumor biology that Black women experience. Importantly, our data show that neighborhood deprivation may influence breast cancer outcomes through downregulation of *LRIG1* because this tumor suppressor gene is a disease survival–associated gene.

In our study, a higher proportion of Black patients with breast cancer lived in neighborhoods with higher deprivation; these same individuals with breast cancer also experienced a higher proportion of TNBC. These findings are suggestive of the role that decades of systemic racism, neighborhood disinvestment,^22^ and socioeconomic deprivation may play in perpetuating breast cancer inequities for Black women.^13,36,37,38^ A recent study investigating DNA methylation and individual neighborhood-level factors found 26 CpG sites to be associated with job density or college education and with all-cause mortality.^12^ These results, combined with our findings, suggest that racism, as experienced through socioeconomic deprivation, may play a part in the poorer health outcomes experienced by Black populations.^39,40,41^

*LRIG1* and *WWOX*, 2 known tumor suppressor genes, were both hypomethylated in tumors of the high neighborhood deprivation patient group. *LRIG1* biologically functions as a negative regulator of receptor tyrosine kinase signaling^42,43^ and has been shown to repress tumor development and growth by antagonizing gene expression of receptor tyrosine kinases, such as ErbB.^44,45,46^ Low *LRIG1* gene expression is considered an independent risk factor for breast cancer metastasis and has been found to be an estrogen-regulated growth suppressor.^47,48^ In our study, *LRIG1* methylation in the gene body region was decreased for patients in the high neighborhood deprivation group. In a cancer context, gene bodies have been shown to lose DNA methylation and become hypomethylated compared with methylation in normal tissue, resulting in decreased transcription of these genes in the tumor tissue.^49,50^ This association would suggest that *LRIG1* expression is decreased in the high neighborhood deprivation patient group, a conclusion further supported by our RNA sequencing gene expression results. Our data also suggest that this loss of *LRIG1* expression may be negatively associated with disease survival.

The tumor suppressor gene *WWOX* impacts cell differentiation, apoptosis, and cell growth. It is encoded by the *FRA16D* locus,^51,52^ one of the most common active chromosomal fragile sites in cancer. This locus is particularly susceptible to loss of heterozygosity, leading to a reduction in *WWOX* gene expression.^51,53,54,55^ Additionally, *WWOX* is a common target for interindividual copy number variation among racial groups,^56^ and its decreased expression has been seen more frequently in individuals with TNBC.^57^ We observed significantly decreased gene expression for *WWOX* in Black women, suggesting a potential association between neighborhood-level risk factors and loss of heterozygosity in *WWOX*.

### Strengths and Limitations

The diversity of our patient cohort is a strength of this study. Additionally, our data set has detailed socioeconomic and clinical participant data and robust DNA methylation and gene expression values that are linked to ecologic neighborhood data. We applied a widely used deprivation measure to capture poverty and structural racism at the neighborhood level. We also adjusted for or stratified by key variables known to be potential confounders in our analysis, including age, self-reported race, and tumor purity. Our study also has some limitations, including (1) potential neighborhood selection bias^58^ due to convenience sampling and missing residential information; (2) inability to make causal inferences due to the cross-sectional study design; (3) potential inadequate control of confounding variables, especially genetic factors related to ancestry that we could not explore; and (4) lack of residential history and information on cumulative lifetime exposures as they relate to neighborhood-level factors.

## Conclusions

The findings from this cross-sectional study reveal novel insights into an association between neighborhood-level socioeconomic disadvantage and breast tumor biology through altered DNA methylation patterns and immune response. Continued investment in public health interventions and policy changes at the neighborhood level are needed to remedy the biological alterations that could make minoritized populations more susceptible to chronic diseases, such as cancer.

## Notes

Supplement 1.
eMethods.

eReferences
**eFigure 1.** Neighborhood Deprivation Index Scores for Hospital Controls and Patients With Breast Cancer in the NCI-Maryland Breast Cancer Study **eTable 1.** Characteristics of NCI-Maryland Breast Cancer Study Participants by Neighborhood Deprivation Status **eFigure 2.** Association of Neighborhood Deprivation Index With Patient Group and Breast Cancer Molecular Subtype **eFigure 3.** Correlation Analysis Between Methylation β-Values and Transcript Levels for *LRIG1* and *WWOX* **eTable 2.** Linear Correlation Coefficients for the Relationship Between Neighborhood Deprivation and Either DNA Methylation in or Gene Expression of *LRIG1* and *WWOX* With Stratification by Self-Reported Race **eFigure 4.** Association of *LRIG1* Expression in Breast Tumors With Relapse-Free and Overall Patient Survival in Stratified Analyses Using the KM Plotter Webtool **eFigure 5.** Methylation and Gene Expression Status of *WWOX* According to Neighborhood Deprivation With the Neighborhood Deprivation Index Coded as a Continuous Variable **eFigure 6.** Relationship Between Immune Cell Subpopulations and Neighborhood Deprivation in Breast Tumor Tissue Using MethylCIBERSORT

Supplement 2.
Data Sharing Statement
