# Supplementary material for: Neighborhood Deprivation and DNA Methylation and Expression of Cancer Genes in Breast Tumors
Source: JAMA Netw Open. 2023 Nov 6;6(11):e2341651. doi: 10.1001/jamanetworkopen.2023.41651 (PMC10628736; doi:10.1001/jamanetworkopen.2023.41651)
Supplement: Supplement 1. — eMethods. eReferences eFigure 1. Neighborhood Deprivation Index Scores for Hospital Controls and Patients With Breast Cancer in the NCI-Maryland Breast Cancer Study eTable 1. Characteristics of NCI-Maryland Breast Cancer Study Participants by Neighborhood Deprivation Status eFigure 2. Association of Neighborhood Deprivation Index With Patient Group and Breast Cancer Molecular Subtype eFigure 3. Correlation Analysis Between Methylation β-Values and Transcript Levels for LRIG1 and WWOX eTable 2. Linear Correlation Coefficients for the Relationship Between Neighborhood Deprivation and Either DNA Methylation in or Gene Expression of LRIG1 and WWOX With Stratification by Self-Reported Race eFigure 4. Association of LRIG1 Expression in Breast Tumors With Relapse-Free and Overall Patient Survival in Stratified Analyses Using the KM Plotter Webtool eFigure 5. Methylation and Gene Expression Status of WWOX According to Neighborhood Deprivation With the Neighborhood Deprivation Index Coded as a Continuous Variable eFigure 6. Relationship Between Immune Cell Subpopulations and Neighborhood Deprivation in Breast Tumor Tissue Using MethylCIBERSORT [file jamanetwopen-e2341651-s001.pdf]

Supplemental Online Content

Jenkins BD, Rossi E, Pichardo C, et al. Neighborhood deprivation and DNA methylation and expression of cancer genes in breast tumors. *JAMA Netw Open*. 2023;6(11):e2341651. doi:10.1001/jamanetworkopen.2023.41651

eMethods
eReferences
eFigure 1. Neighborhood Deprivation Index Scores for Hospital Controls and Patients With Breast Cancer in the NCI-Maryland Breast Cancer Study
eTable 1. Characteristics of NCI-Maryland Breast Cancer Study Participants by Neighborhood Deprivation Status
eFigure 2. Association of Neighborhood Deprivation Index With Patient Group and Breast Cancer Molecular Subtype
eFigure 3. Correlation Analysis Between Methylation β-Values and Transcript Levels for *LRIG1* and *WWOX*
eTable 2. Linear Correlation Coefficients for the Relationship Between Neighborhood Deprivation and Either DNA Methylation in or Gene Expression of *LRIG1* and *WWOX* With Stratification by Self-Reported Race
eFigure 4. Association of *LRIG1* Expression in Breast Tumors With Relapse-Free and Overall Patient Survival in Stratified Analyses Using the KM Plotter Webtool
eFigure 5. Methylation and Gene Expression Status of *WWOX* According to Neighborhood Deprivation With the Neighborhood Deprivation Index Coded as a Continuous Variable
eFigure 6. Relationship Between Immune Cell Subpopulations and Neighborhood Deprivation in Breast Tumor Tissue Using MethylCIBERSORT

This supplemental material has been provided by the authors to give readers additional information about their work.

© 2023 Jenkins BD et al. *JAMA Network Open.*

eMethods

NCI-Maryland Breast Cancer Study

The National Cancer Institute (NCI)-Maryland Breast Cancer study was conducted among women who self-reported as Black or White. Recruits included women undergoing breast surgery who did or did not have a diagnosis of breast cancer. 456 total participants were recruited into the study mainly between January 1, 1992, and January 1, 2003 (82%). A small subset of additional participants were recruited after 2003 up until January 11, 2019 (18%). 597 total tissue specimens were collected. For this study, we excluded:

1. n = 38 male samples. Study was restricted to females only.

2. n = 2 Asian samples, n = 2 Hispanic samples. Due to small sample size of other races/ethnicities, study was restricted to Black or White participants only.

3. n = 143 adjacent normal breast tissue samples and n=104 normal breast tissue samples (hospital controls). Study was restricted to tumor tissue only.

4. n = 90 autopsy/normal samples. No exposure data was available as geocoding was not possible for these samples. Study was restricted to samples with available exposure data.

5. n = 32 samples without residential information or geocoding consent. Study was restricted to samples with available exposure data.

6. n = 1 sample that did not pass methylation quality control

Our final analytic population included 185 participants who donated breast tumor tissue.

The collection of biospecimens and the clinical and pathological information was approved by the UMD Institutional Review Board (IRB) for the participating institutions (UMD protocol #0298229). IRB approval of this protocol was then obtained at all institutions with a collection site (Baltimore Veterans Affairs Medical Center, Union Memorial Hospital, Mercy Medical Center, and Sinai Hospital). The research was also reviewed and approved by the NIH Office of Human Subjects Research Protections (OHSRP #2248).

Race Categorization

Race was self-reported by study participants, and race categories were defined by investigators based on the US Office of Management and Budget's Revisions to the Standards for the Classification of Federal Data on Race and Ethnicity. Data for this study included U.S. adults who self-reported as non-Hispanic Black (hereafter, Black), and non-Hispanic White (hereafter, White) individuals. Due to the social and economic implications of this work, terms describing race as a social construct (such as Black or White) were used throughout.

Neighborhood Deprivation Index

A principal components analysis was used for data reduction based on a study that validated the index in Maryland, USA[1]. The shared variance from the 20 variables representing the socioeconomic position of the study participants were reduced to a single factor using variable loadings[2]. Loadings above 0.25 were retained without further consideration for the 95% confidence limit for each loading. Clustering patterns were assessed by Census tract and showed that the participants were drawn from across all tracts, with most tracts having only one participant, limiting any bias by geographic location.

Quality Control of DNA Methylation Data

Further QC analysis was done using the Methylaid package in R to detect any poor-quality samples. Doing so, we identified one tumor sample as an outlier, which was then removed from further analysis. Batch effects were

© 2023 Jenkins BD et al. *JAMA Network Open.*

adjusted for using the B Clear and ComBat packages in R. Promoter and gene body-specific DNA methylation was measured by determining the median beta value for all CpG sites in either the promoter or gene body region for a particular gene of interest.

Tumor Purity Variable

Tumor purity was assessed using the *InfinumPurify* R package, which uses a methylation beta value matrix from tumor samples and tumor type as inputs and outputs a tumor purity vector for all tumor samples[3]. Hospital controls (n=104) were used as the training group and tumor purity in our samples were found to be within normal range compared with other published work. As a result, all samples were kept after tumor purity assessment.

RNA-Seq Pre-processing and Analysis

RNA was extracted from breast tumor tissue and one µg was sent to the Sequencing Facility in the Center for Cancer Research at NCI for library preparation using the TruSeq PolyA kit (Illumina, San Diego, California, USA). Sequencing was performed with the Nova Seq system using 150 bp paired end reads. *Trimmomatic* software was used to trim reads and about 90% uniquely mapped to the human genome (hg38) using *STAR*. RNA sequencing (RNA-Seq) mapping statistics were calculated using *Picard*, which showed 90% of the reads being successfully mapped to the transcriptome. *HTSeq* software was used to calculate read count per gene under the Gencode annotation. Data was normalized by size factor using the *DESeq2* package. Further gene expression analysis used regularized-logarithm transformation (rlog) values and compared these to continuous neighborhood deprivation measurements.

Kaplan-Meier Survival Analysis

Kaplan-Meier (KM) plots estimating relapse-free and overall survival related to gene expression in tumors were generated using https://kmplot.com with gene chip-derived and RNA-Seq-derived data from The Cancer Genome Atlas (TCGA) for women with breast cancer. High and low gene expression value cutoffs were generated using the median as the cutoff for dichotomization. The 211596_s_at probe set was used for estimating *LRIG1* expression when gene chip-derived gene expression data were used.

eReferences

1 Salmon, C. *et al.* Neighbourhood social deprivation and risk of prostate cancer. *Br J Cancer* (2023). https://doi.org:10.1038/s41416-023-02299-7

2 Pichardo, M. S. *et al.* Association of Neighborhood Deprivation With Prostate Cancer and Immune Markers in African American and European American Men. *JAMA Netw Open* **6**, e2251745 (2023). https://doi.org:10.1001/jamanetworkopen.2022.51745

3 Qin, Y., Feng, H., Chen, M., Wu, H. & Zheng, X. InfiniumPurify: An R package for estimating and accounting for tumor purity in cancer methylation research. *Genes Dis* **5**, 43-45 (2018). https://doi.org:10.1016/j.gendis.2018.02.003

© 2023 Jenkins BD et al. *JAMA Network Open.*

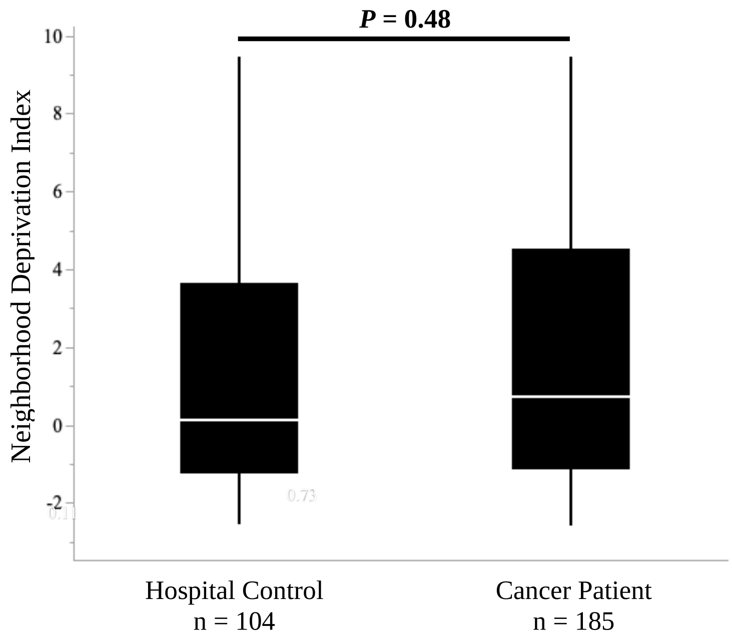

eFigure 1. Neighborhood Deprivation Index Scores for Hospital Controls and Patients With Breast Cancer in the NCI-Maryland Breast Cancer Study. Box plots depicting median and quantile differences in the NDI scores comparing hospital controls (n = 104) with breast cancer patients (n = 185). Hospital controls were women with breast reduction surgery and were cancer-free.

© 2023 Jenkins BD et al. *JAMA Network Open.*

eTable 1. Characteristics of NCI-Maryland Breast Cancer Study Participants by Neighborhood Derivation Status

Demographics	Patients (n=185)		
	Low[a] Neighborhood Deprivation (n=90)	High Neighborhood Deprivation (n=95)	*P* value[b,c]
Age[d]			
Mean (SD) in years	53.9 (13.4)	58.1 (14.6)	**0.05**
BMI			
Mean (SD) in kg/m^2	28.6 (7.3)	30.5 (6.8)	n.s.
Self-reported race, *No.* (%)			
Black/African American	31 (34)	79 (83)	**<0.001**
White/European American	59 (66)	16 (17)	
Missing	0	0	
Education, *No.* (%)			
Less than high school	8 (10)	31 (36)	**<0.001**
High school graduate	46 (57)	44 (52)	
College Degree	13 (16)	6 (7)	
Graduate School	14 (17)	4 (5)	
Missing	9	10	
Annual household income, *No.* (%)			
≤ $60,000	50 (68)	71 (91)	**<0.001**
> $60,000	24 (32)	7 (9)	
Missing	16	17	
Marital Status, *No.* (%)			
Never married	13 (17)	33 (38)	**<0.001**
Married	39 (51)	21 (24)	
Divorced/Widowed/ Separated	25 (32)	34 (39)	
Missing	13	7	
**Health Factors**			
Ever Smoker[e], *No.* (%)			
No	33 (56)	34 (49)	n.s.
Yes	26 (44)	35 (51)	
Missing	31	26	
Alcohol Use, *No.* (%)			
No	20 (34)	34 (49)	n.s.
Yes	38 (66)	36 (51)	
Missing	32	25	
Diabetes, *No.* (%)			
No	51 (86)	53 (73)	**0.05**
Yes	8 (14)	20 (27)	
Missing	31	22	
Family History of Breast Cancer, *No.* (%)			
No	44 (77)	57 (83)	n.s.
Yes	13 (23)	12 (17)	
Missing	33	26	
Menopause, *No.* (%)			
Pre-menopausal	19 (40)	18 (28)	n.s.

© 2023 Jenkins BD et al. *JAMA Network Open.*

Post-menopausal	29 (60)	46 (72)	
Missing	42	31	
**Clinical Factors**			
Grade, *No.* (%)			
1	8 (16)	4 (6)	**0.03**
2	23 (46)	20 (32)	
3	19 (38)	38 (61)	
Missing	40	33	
Stage[f], *No.* (%)			
I	13 (15)	17 (18)	n.s.
II	48 (55)	54 (57)	
III	19 (22)	19 (20)	
IV	1 (1)	1 (1)	
TIS	7 (8)	3 (3)	
Missing	2	1	
Molecular Subtype, *No.* (%)			
HR+	49 (64)	40 (49)	n.s.
HER2+	11 (14)	14 (17)	
TNBC	17 (22)	28 (34)	
Missing	13	13	
NACT[g], *No.* (%)			
No	33 (60)	47 (70)	n.s.
Yes	22 (40)	20 (30)	
Missing	35	28	

Abbreviations: SD, standard deviation, BMI, body mass index, n.s., not significant

[a] Dichotomization of neighborhood deprivation was based on the median index values (≤ median vs. > median) in non-cases (see Methods, eFigure 1 in Supplement 1)

[b] *P* values for continuous variables determined by Student's t-test

[c] *P* values for categorical variables determined by Chi-squared test, bolded *P* values significant at $P < 0.05$

[d] Age at surgery

[e] Smoking status describes cigarette smoking

[f] Pathologically confirmed using American Joint Committee on Cancer (AJCC)

[g] Neoadjuvant chemotherapy

© 2023 Jenkins BD et al. *JAMA Network Open.*

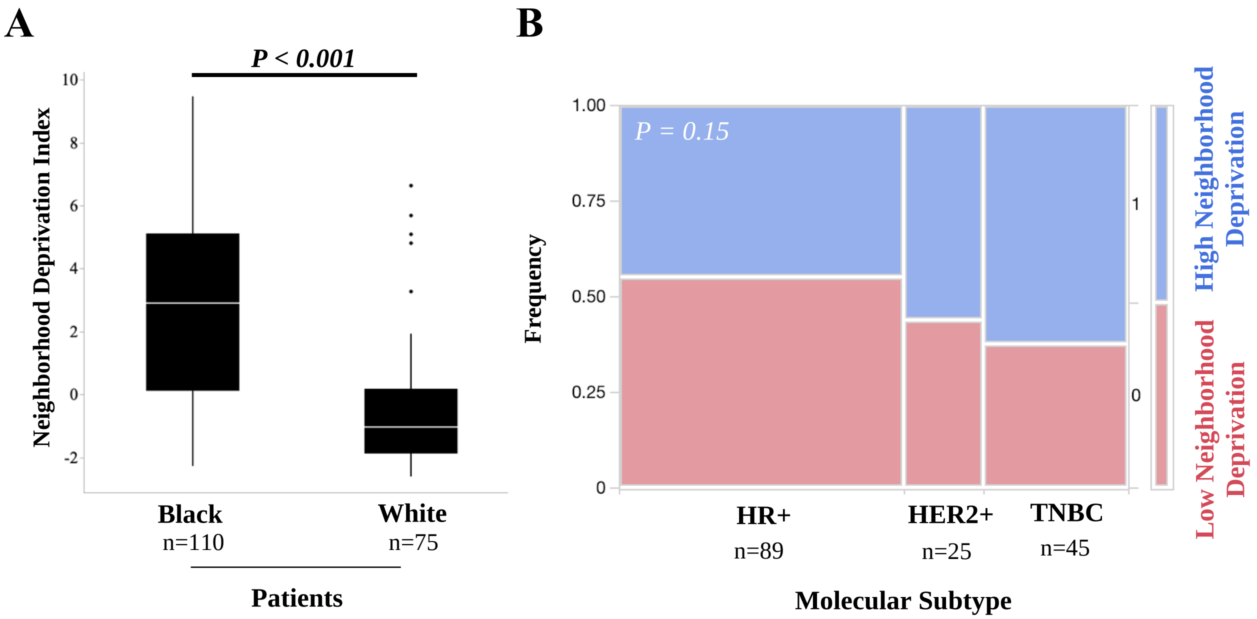

eFigure 2. Association of Neighborhood Deprivation Index With Patient Group and Breast Cancer Molecular Subtype. (A) Neighborhood deprivation as a continuous variable is higher among Black than White patients. Welch's t-test. **(B)** Mosaic plot capturing the association of dichotomized neighborhood deprivation with breast cancer molecular subtypes. HR, Hormone Receptor; HER2, Human Epidermal Growth Factor Receptor 2; TNBC, Triple Negative Breast Cancer. Chi-squared test.

© 2023 Jenkins BD et al. *JAMA Network Open.*

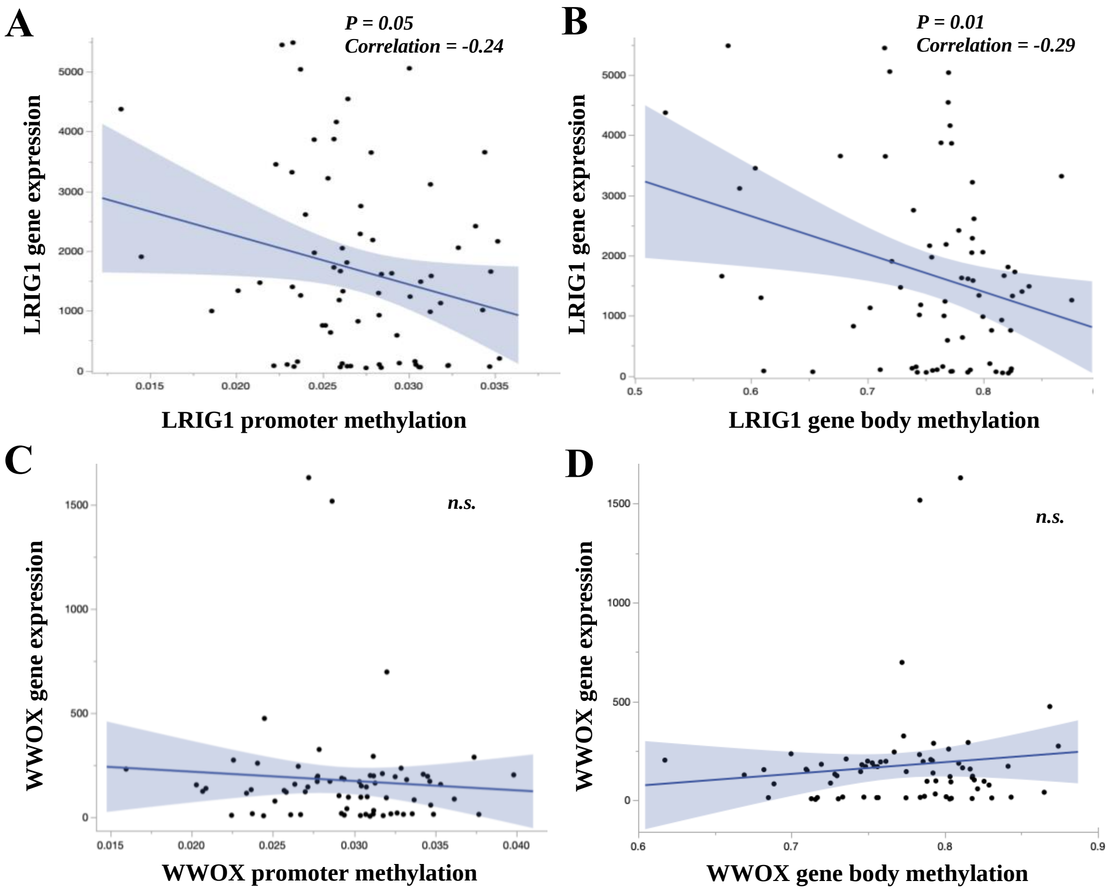

eFigure 3. **Correlation Analysis Between Methylation β-Values and Transcript Levels for *LRIG1* and *WWOX*.** **(A)** Fit line represents the correlation between promoter methylation and RNA-Seq-based gene expression values for *LRIG1*. (B) same at (A) but with *LRIG1* gene body methylation and *LRIG1* gene expression. (C) Correlation between promoter methylation and RNA-Seq-based gene expression values for *WWOX*. (D) Same as (C) but with *WWOX* gene body methylation. *P* values are derived from the analysis of variance tests using continuous variables. n.s., not significant.

© 2023 Jenkins BD et al. *JAMA Network Open.*

eTable 2. Linear Correlation Coefficients for the Relationship Between Neighborhood Deprivation and Either DNA Methylation in or Gene Expression of *LRIG1* and *WWOX* With Stratification By Self-Reported Race

		**Black**			**White**		
		**Neighborhood Deprivation**			**Neighborhood Deprivation**		
**Gene**	**Data source**	**Correlation Coefficient**	**95% CI**	***P* value[a]**	**Correlation Coefficient**	**95% CI**	***P* value[a]**
LRIG1	Methylation (cg26131019)	-0.203	-0.376, -0.017	**0.03**	-0.062	-0.285, 0.167	0.60
LRIG1	Gene Expression (RNA-Seq)	-0.390	-0.613, -0.109	**0.008**	-0.103	-0.472, 0.296	0.62
WWOX	Methylation (cg26131019)	-0.149	-0.328, -0.039	0.12	-0.189	-0.399, 0.039	0.10
WWOX	Gene Expression (RNA-Seq)	-0.433	-0.645, -0.160	**0.003**	-0.111	-0.478, 0.590	0.59

Abbreviations: CI, confidence interval

[a]*P* values derived from Pearson's correlation coefficient, bolded *P* values are significant at $P < 0.05$

© 2023 Jenkins BD et al. *JAMA Network Open.*

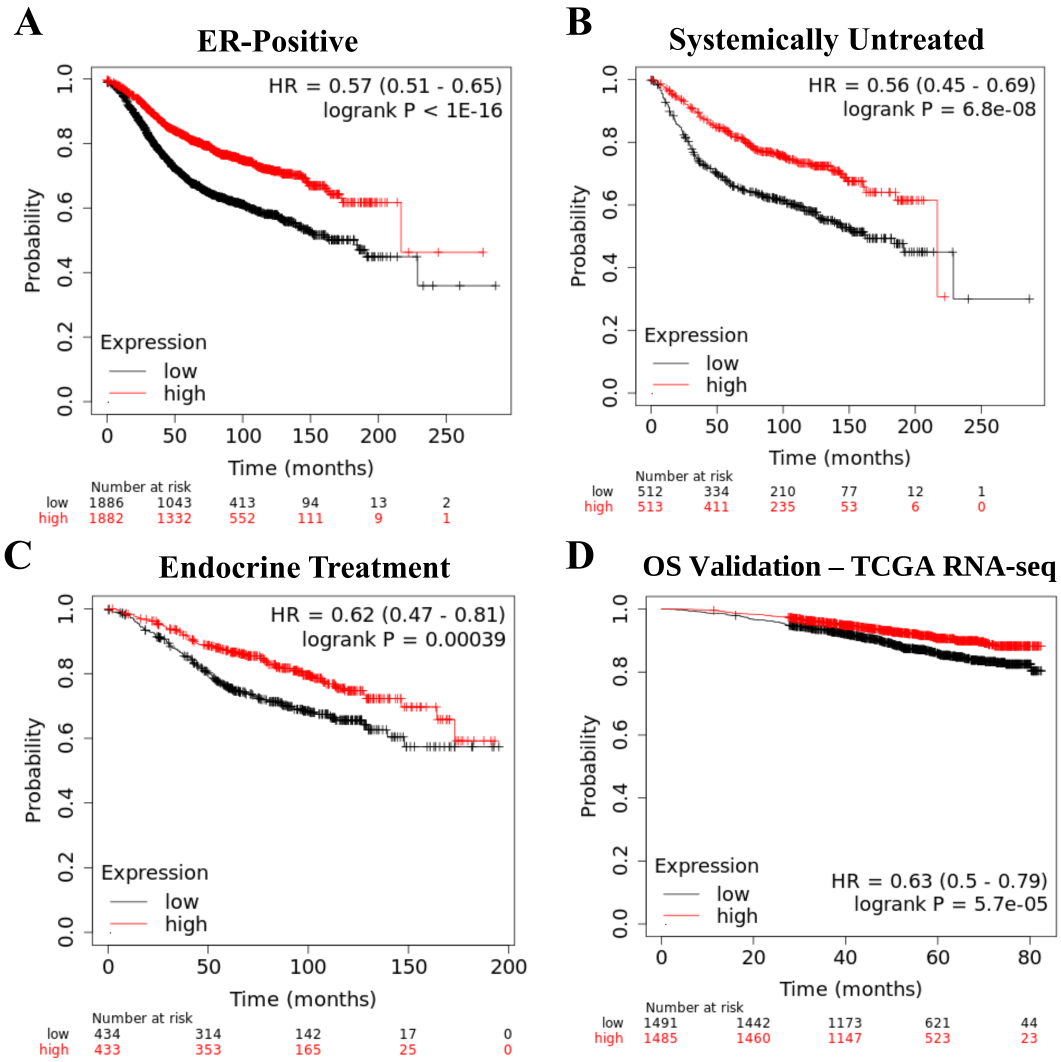

eFigure 4. Association of *LRIG1* Expression in Breast Tumors With Relapse-Free and Overall Patient Survival in Stratified Analyses Using the KM Plotter Webtool. Kaplan-Meier plots depicting relapse-free breast cancer survival by median dichotomized *LRIG1* expression (Gene chip probe 211596_s_at) for women with (**A**) estrogen-receptor positive tumors (n = 3,768), (**B**) systemically untreated tumors (n = 1,025), or (**C**) tumors treated with adjuvant endocrine therapy (n = 867). (**D**) Overall survival (OS) using a TCGA RNA-seq-based validation dataset provided by the KM plotter webtool (see eMethods). Black lines indicate low gene expression and red lines indicate high gene expression.

© 2023 Jenkins BD et al. *JAMA Network Open.*

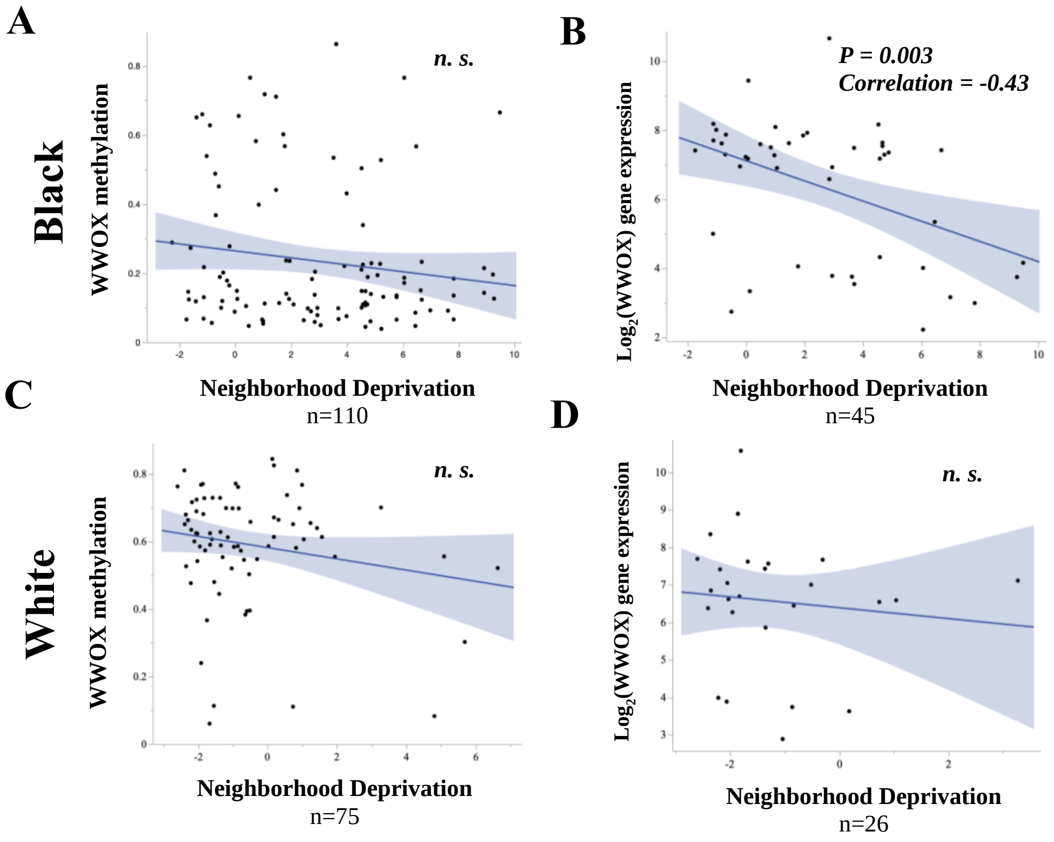

eFigure 5. Methylation and Gene Expression Status of *WWOX* According to Neighborhood Deprivation With the Neighborhood Deprivation Index Coded as a Continuous Variable. Linear regression model showing methylation beta values for *WWOX* (cg02171206) by continuous neighborhood deprivation in **(A)** Black and **(C)** White women. Linear regression model showing log_2-transformed gene expression data from RNA-Seq for *WWOX* by continuous neighborhood deprivation in **(B)** Black women and **(D)** White women. n.s., not significant.

© 2023 Jenkins BD et al. *JAMA Network Open.*

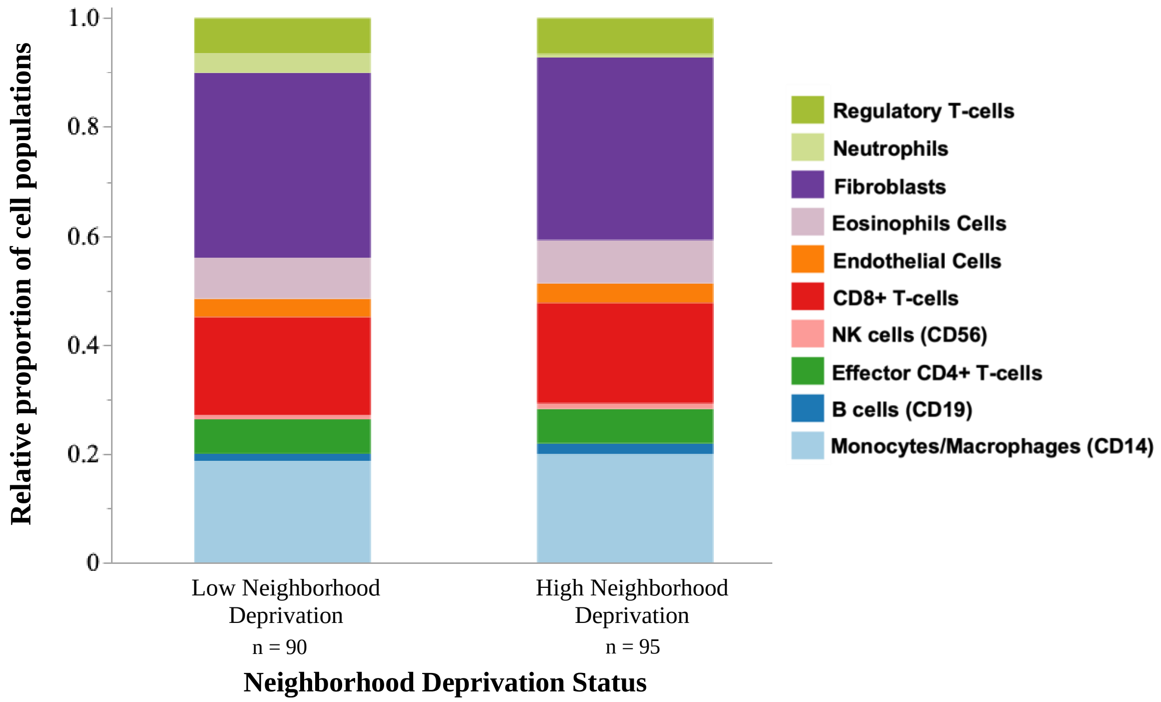

eFigure 6. Relationship Between Immune Cell Subpopulations and Neighborhood Deprivation in Breast Tumor Tissue Using MethylCIBERSORT. Relative immune cell sub-population scores are compared with dichotomized neighborhood deprivation in 185 breast tumors.

© 2023 Jenkins BD et al. *JAMA Network Open.*
