# Supplementary material for: Neighborhood Deprivation and DNA Methylation and Expression of Cancer Genes in Breast Tumors
Source: JAMA Netw Open. 2023 Nov 6;6(11):e2341651. doi: 10.1001/jamanetworkopen.2023.41651 (PMC10628736; doi:10.1001/jamanetworkopen.2023.41651)
Supplement: Supplement 2. — Data Sharing Statement [file jamanetwopen-e2341651-s002.pdf]

Data Sharing Statement

Jenkins. Neighborhood Deprivation and DNA Methylation and Expression of Cancer Genes in Breast Tumors. *JAMA Netw Open*. Published November 07, 2023.
doi:10.1001/jamanetworkopen.2023.41651

Data
Data available: No

Additional Information
Explanation for why data not available: Clinical, demographic and methylation data were deposited in the NCBI's Gene Expression Omnibus (GEO) database under accession number GSE225845 and is publicly available. The RNAseq data for the human breast tumors were deposited in the NCBI's Gene Expression Omnibus (GEO) database under accession number GSE225846 and is also publicly available. The remaining data are available within the article, or as supplementary data, or are available from the authors upon request. Personal identifiers such as neighborhood census tract data cannot be shared.
